# Adjuvant-Enabled Halving of Florpyrauxifen-Benzyl Dose Maintains Paddy Weed Control and Enhances Soil Health and Rice Yield

**DOI:** 10.3390/plants15111688

**Published:** 2026-05-29

**Authors:** Yuan Gao, Huifeng Wang, Jiapeng Fang, Guohui Yuan, Zhihui Tian, Lirong Wang

**Affiliations:** 1 Eco-Environmental Protection Research Institute, Shanghai Academy of Agricultural Sciences, Shanghai 201403, China; gaoyuan@saas.sh.cn (Y.G.); 20220701@saas.sh.cn (J.F.); yuanguohui@saas.sh.cn (G.Y.); 2 Faculty of Chemical Engineering and Energy Technology, Shanghai Institute of Technology, Shanghai 201418, China; 256061130@sit.edufp.cn

**Keywords:** barnyardgrass, herbicide reduction, synergistic effect, herbicide residue, soil microbial community, soil physicochemical properties, sustainable rice production

## Abstract

Reducing herbicide input in paddy fields is essential for sustainable rice production and long-term soil health. Florpyrauxifen-benzyl effectively controls the dominant paddy weed barnyardgrass (*Echinochloa crus-galli*), yet excessive application poses environmental risks. Here, we investigated whether the compound adjuvant Sijiling, containing nonionic and anionic surfactants, could enable significant dose reduction in florpyrauxifen-benzyl while maintaining weed control efficacy and improving soil–plant system functions. Greenhouse dose–response assays and two-year field trials conducted in 2021 and 2022 demonstrated that the adjuvant permitted a 50% reduction in herbicide application without compromising control of barnyardgrass or other paddy weeds. Mechanistically, Sijiling disrupted the leaf cuticular wax barrier and amplified ethylene and ABA biosynthesis over two-fold. The reduced herbicide rate lowered residues in rice and soil, increased soil organic carbon and available potassium, and enhanced microbial diversity, particularly enriching beneficial Acidobacteria. Grain yield increased significantly under the reduced-input strategy, with Mantel analysis linking yield gains to improved soil available potassium and organic carbon. Our findings demonstrate that adjuvant-enabled herbicide dose reduction is an effective and sustainable weed management strategy for paddy rice, maintaining robust weed suppression while delivering measurable co-benefits for soil health and crop productivity, thereby supporting the sustainable intensification of rice-based cropping systems.

## 1. Introduction

Rice (*Oryza sativa* L.) is the staple food for more than half of the global population, and its sustainable production is fundamental to food security. Weeds, particularly barnyardgrass (*Echinochloa crus-galli* (L.) Beauv.), represent a major constraint in paddy rice cultivation, causing substantial yield losses [1,2,3,4]. Effective weed management is therefore essential for maintaining rice productivity and ensuring the economic viability of paddy farming systems. Chemical herbicides remain the most widely adopted tool for weed control in paddy fields. Florpyrauxifen-benzyl is a highly effective auxin-type herbicide against resistant barnyardgrass biotypes [5,6]. In China, it is among over 40 herbicidal active ingredients registered for rice, with a recommended application rate ranging from 18 to 36 g a.i. ha^−1^ [7]. However, due to its favorable safety profile in rice beyond the 4.0-leaf stage, many farmers apply the maximum dosage or even exceed it, particularly in heavily infested paddy fields. Our field interviews in Shanghai, China revealed that some farmers applied florpyrauxifen-benzyl at approximately 54 g a.i. ha^−1^, 1.5 times the maximum recommended rate of 36 g a.i. ha^−1^.

Excessive herbicide use can first cause direct phytotoxicity to crops, affecting both the target rice field (Supplementary Figure S1) and adjacent croplands (Supplementary Figure S2). Moreover, it not only raises production costs but also poses potential risks to soil health and ecosystem functioning in paddy agroecosystems. Accumulating evidence indicates that high herbicide inputs and overdosing can alter soil physicochemical properties, suppress beneficial microbial communities, and leave residues that may affect non-target organisms and subsequent crops [8,9,10,11]. In paddy soils, health is intimately linked to crop productivity through complex plant–soil feedbacks, where soil organic carbon, nutrient availability, and microbial diversity play pivotal roles [12,13]. Therefore, strategies that reduce herbicide application without compromising paddy weed control are urgently needed to maintain soil functionality and ensure long-term rice production.

One promising approach is the use of spray adjuvants, which are added to herbicide solutions to modify physicochemical properties and improve herbicide deposition, spreading, and penetration [14,15]. Adjuvants can enhance herbicide efficacy, allowing dose reduction while maintaining weed control [16]. However, the chemical specificity of adjuvant–herbicide interactions means that not all adjuvants are effective with a given herbicide [17]. Moreover, while adjuvants are increasingly used in crop protection, their environmental implications—particularly how adjuvant-enabled dose reduction affects paddy soil properties and microbial communities—remain largely unexplored. To date, no systematic investigation has addressed adjuvant screening for florpyrauxifen-benzyl in barnyardgrass management, nor has any study comprehensively evaluated the plant–soil consequences of such reduced-input strategies in paddy rice production systems.

We hypothesized that the compound adjuvant Sijiling could synergistically enhance florpyrauxifen-benzyl efficacy, enabling a substantial reduction in herbicide application without compromising barnyardgrass control, and that this reduced-input strategy would yield co-benefits for soil health and rice productivity. To test this hypothesis, we aimed to identify a compatible adjuvant for florpyrauxifen-benzyl and determine the optimal synergistic ratio that allows herbicide dose reduction in paddy fields. We then sought to elucidate the mechanistic basis of any observed synergy, focusing on leaf cuticular wax disruption and the biosynthesis of the phytotoxic hormones ethylene and abscisic acid. Finally, we assessed the environmental and agronomic outcomes of the optimized combination, including herbicide residues, paddy soil physicochemical properties, soil microbial community composition and diversity, and ultimately rice yield. By integrating chemical, plant physiological, and soil biological perspectives, this work provides a comprehensive understanding of how adjuvant-assisted herbicide dose reduction can sustain paddy weed control while enhancing soil health and crop productivity—a finding with direct implications for sustainable rice production and integrated weed management in paddy fields.

## 2. Results

### 2.1. Synergistic Effect of Sijiling with Florpyrauxifen-Benzyl

Among seven commercial pesticide spray adjuvants evaluated for enhancing florpyrauxifen-benzyl efficacy against barnyardgrass, three adjuvants—Sijiling, Meidun, and Jijian—demonstrated significant synergistic effects; Huoniu and Hasuteng exhibited antagonistic effects, while Maisi and Jiadeli showed no significant impact. Three adjuvants demonstrated significant synergy (*p* < 0.05): Sijiling (V2, V3 ratios), Meidun (V2, V3), and JJ (V1, V2). Sijiling at V3 ratio showed the strongest enhancement, increasing weed control efficacy by approximately 16% (Table 1; Supplementary Figure S3). Additionally, Sijiling exhibited no universal synergistic effects with other common rice herbicides (metamifop, pyraquinate, propanil) (Supplementary Figure S4).

**Figure 1:**
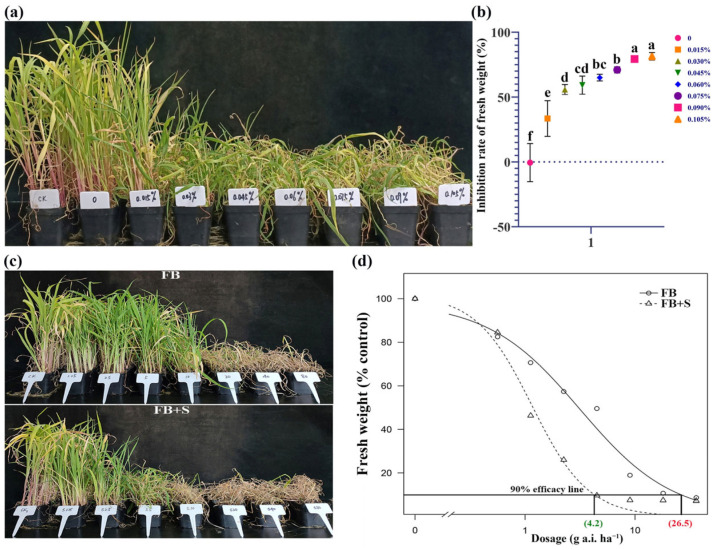
Screening and validation of the optimal addition ratio of the Sijiling adjuvant. (**a**) The inhibitory effect of the combinations of Sijiling at different addition ratios and florpyrauxifen-benzyl on barnyardgrass (at 4.0-leaf stage). (**b**) A comparison of the inhibition rate of fresh weight of barnyardgrass through treatment with combinations of different addition ratios of Sijiling and florpyrauxifen-benzyl. (**c**) The difference in the inhibitory effects of florpyrauxifen-benzyl alone and the combination of 0.09% Sijiling + florpyrauxifen-benzyl on barnyardgrass. (**d**) The effect of adding 0.09% Sijiling on the toxicity of florpyrauxifen-benzyl in controlling barnyardgrass. FB: Florpyrauxifen-benzyl; S: Sijiling.

We evaluated the synergistic effect of Sijiling adjuvant across varying concentrations combined with florpyrauxifen-benzyl for barnyardgrass control. Florpyrauxifen-benzyl applied alone at 3.6 g a.i. ha^−1^ showed no significant herbicidal activity against 4.0-leaf stage barnyardgrass. However, the addition of Sijiling at 0.015–0.105% (*v*/*v*) demonstrated significant dose-dependent synergy, with fresh weight inhibition increasing progressively with adjuvant concentration. Maximum efficacy was achieved at 0.09% and 0.105% Sijiling, with no statistical difference between these two concentrations (Figure 1a,b). Dose–response analysis revealed that the GR_50_ and GR_90_ values for florpyrauxifen-benzyl alone were 3.2 and 26.5 g a.i. ha^−1^, respectively. The addition of 0.09% Sijiling substantially reduced these values to 1.2 (GR_50_) and 4.2 (GR_90_) g a.i. ha^−1^ (Figure 1c,d). This represents a 62.5% reduction in florpyrauxifen-benzyl requirement for 50% fresh weight inhibition and an 84.2% reduction for 90% inhibition in 2.5–3.0 leaf stage barnyardgrass.

**Figure 2:**
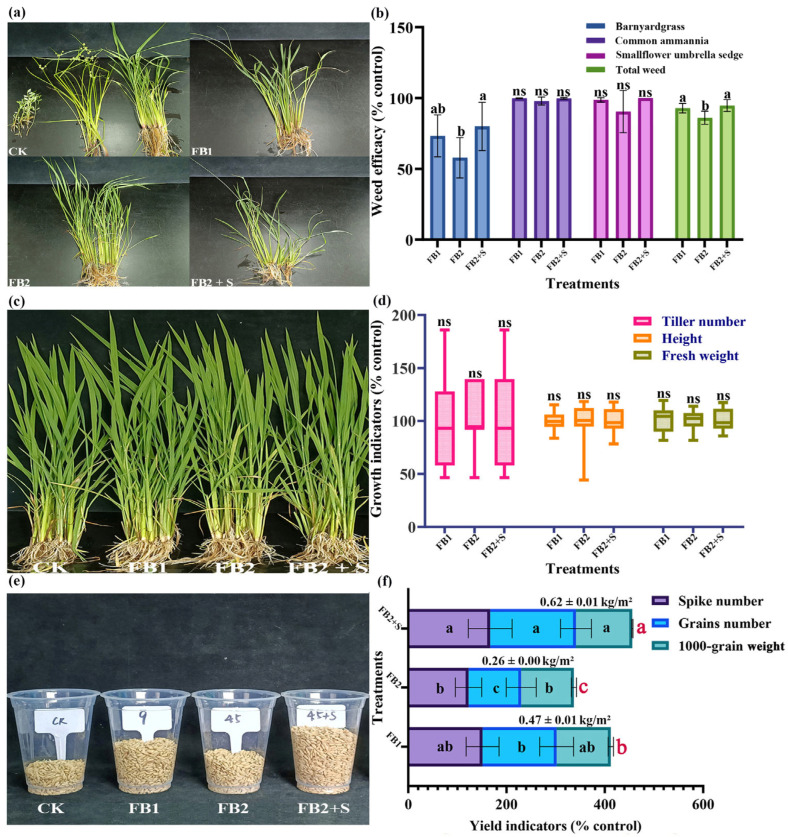
The efficacy and selectivity of the combination of florpyrauxifen-benzyl and Sijiling. (**a**) The remaining weeds and poisoning symptoms in each treatment area. (**b**) The plant control efficacy of each treatment on weeds. a–b represent the significant difference in this indicator among the three treatments. “ns” indicates no significant difference. (**c**) The growth status of rice in each treatment. (**d**) The differences in rice growth indicators among the different treatments. “ns” indicates no significant difference. (**e**) The amount of rice in one sampling box (0.25 m^2^, 50 cm × 50 cm) in each process. (**f**) The differences in rice yield indicators among the different treatments. The a–c in black font represents the significant difference in this indicator among the three treatments. The a–c in red font represents significant differences in rice yield among the three treatments.

Field experiments validated the efficacy and selectivity of florpyrauxifen-benzyl combined with Sijiling adjuvant. Treatment effects were primarily distinguished in barnyardgrass control (Figure 2a). At 21 days after treatment, FB1 (27.0 g a.i. ha^−1^), FB2 (13.5 g a.i. ha^−1^), and FB2+S (13.5 g a.i. ha^−1^ + 0.09% Sijiling) demonstrated control efficacies of 73.3%, 57.8%, and 80.0%, respectively (*p* < 0.05). All three treatments provided >90% control of the other two naturally occurring weed species—common ammannia (*Ammannia* spp.) and smallflower umbrella sedge (*Cyperus difformis*)—without significant differences. The total weed control efficacy of FB2+S and FB1 significantly surpassed FB2 (*p* < 0.05) (Figure 2b). Selectivity assessment revealed no phytotoxicity to rice, with tiller number, plant height, and fresh weight maintaining 95–105% of control values across treatments (Figure 2c,d). Values slightly exceeding 100% likely reflect reduced weed competition in herbicide-treated plots, which may promote compensatory rice growth. No significant differences were observed among the treatments for any of these parameters (ns, *p* > 0.05). Field validation confirmed that the 50% reduced rate combined with Sijiling (FB2+S) achieved superior total weed control compared to the full rate alone (FB2), without any phytotoxicity to rice (Figure 2a–d). The impact of this optimized treatment on grain yield was further analyzed in relation to soil health indicators (see Section 2.5).

### 2.2. Effects on Leaf Cuticular Wax and Phytotoxic Hormone Biosynthesis

**Figure 3:**
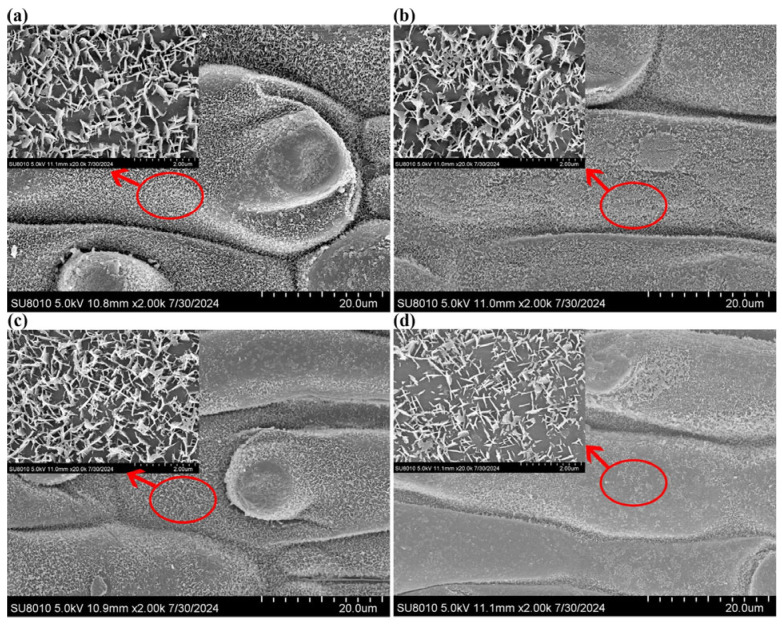
The cryo-SEM images of wax crystal dispersion and structure on the adaxial side of barnyardgrass. (**a**) Barnyardgrass leaf treated with water (CK), (**b**) 13.5 g a.i. ha^−1^ florpyrauxifen-benzyl, (**c**) 27.0 g a.i. ha^−1^ florpyrauxifen-benzyl, and (**d**) 13.5 g a.i. ha^−1^ florpyrauxifen-benzyl+0.09% (*v*/*v*) Sijiling. The scale and electron microscope parameters are shown at the bottom of each image.

Cryo-scanning electron microscopy revealed that Sijiling adjuvant caused marked disruption of the epicuticular wax layer on barnyardgrass leaves. Barnyardgrass leaves typically exhibited dense, spindle-shaped wax crystals (Figure 3a). Treatment with florpyrauxifen-benzyl alone (FB1 and FB2) showed minimal morphological changes compared to the control (Figure 3b,c). However, FB2+S (florpyrauxifen-benzyl+Sijiling) caused significant wax crystal reduction and sparse distribution (Figure 3d). Quantitative analysis of the cryo-SEM images using ImageJ software (version 1.54g) revealed that wax crystal coverage on the adaxial leaf surface was 75.33% in the control, 71.63% in FB2, 82.28% in FB1, and markedly reduced to 43.46% in FB2+S (*p* < 0.05).

We further examined ethylene and abscisic acid biosynthesis, key phytotoxicity mediators for auxin-type herbicides. ACC (1-aminocyclopropane-1-carboxylic acid) accumulation peaked at 12 h, with FB2+S producing 2404.17 ng g^−1^—significantly higher than other treatments and the control (*p* < 0.01). The ACC synthesis hierarchy was FB2+S > FB1 > FB2 > control, with significant intergroup differences (*p* < 0.01). ABA accumulation followed similar patterns: FB2+S > FB1 ≈ FB2 > control. While FB1 and FB2 showed comparable ABA levels, other comparisons revealed significant differences (*p* < 0.01) (Table 2).

### 2.3. Herbicide Residues and Soil Physicochemical Properties

To compare herbicide degradation patterns, florpyrauxifen-benzyl residues in soil and rice were analyzed following application alone at the full rate (FB1: 27.0 g a.i. ha^−1^) or combined with Sijiling adjuvant at the reduced rate (FB2+S: 13.5 g a.i. ha^−1^ + 0.09% Sijiling), both of which provided equivalent barnyardgrass control (Table 3).

**Figure 4:**
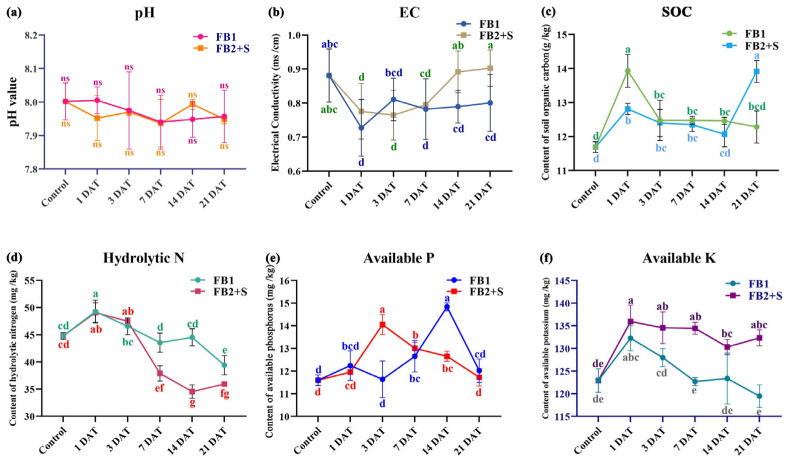
The differences in the physical and chemical properties of soil between FB1 and FB2+S. (**a**) soil pH value, (**b**) soil electrical conductivity, (**c**) soil organic carbon content, (**d**) hydrolytic nitrogen content in soil, (**e**) available phosphorus content in soil, and (**f**) content of available potassium in soil for the different treatment groups. FB1: 27.0 g a.i. ha^−1^ florpyrauxifen-benzyl; FB2+S: 13.5 g a.i. ha^−1^ florpyrauxifen-benzyl + 0.09% (*v*/*v*) Sijiling. “ns” indicates no significant difference. “a–g” indicate significant differences (Duncan’s test, *p* < 0.05).

We analyzed key soil physicochemical properties influencing crop growth: pH, electrical conductivity (EC), soil organic carbon (SOC), hydrolytic nitrogen, available phosphorus, and available potassium. Soil pH remained stable (7.9–8.1) throughout the experimental period with no significant differences between FB1 (florpyrauxifen-benzyl alone) and FB2+S (florpyrauxifen-benzyl + Sijiling) (Figure 4a). Soil EC showed no initial divergence but became significantly higher in FB2+S than FB1 at 14 and 21 days after treatment (DAT) (Figure 4b). At 1 DAT, SOC content was significantly higher in FB1 than in FB2+S (*p* < 0.05), but reversed to higher levels in FB2+S by 21 DAT (Figure 4c). Hydrolytic nitrogen was consistently lower in FB2+S at 7, 14, and 21 DAT (Figure 4d). Available phosphorus exhibited considerable fluctuation, with both treatments alternating in maximum values (Figure 4e). Available potassium was significantly elevated in FB2+S at all post-treatment sampling points (3, 7, 14, 21 DAT) (Figure 4f).

### 2.4. Lower Herbicide Rate Enhances Soil Microbial Diversity and Alters Community Structure

**Figure 5:**
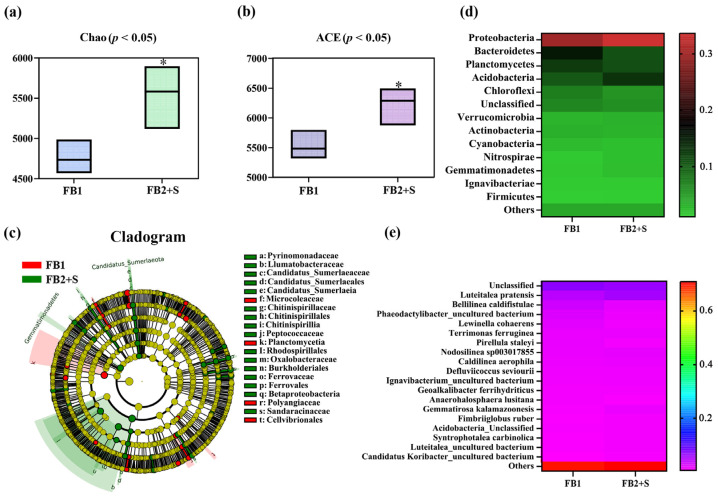
Soil microbial community structure and diversity under FB1 and FB2+S treatments at 21 DAT. (**a**) Chao1 richness index. (**b**) ACE diversity index. (**c**) Cladogram of differentially abundant microbial taxa. The linear discriminant analysis (LDA) threshold was set to 2.0 to identify significantly enriched microbial taxa between the treatments. (**d**) Phylum-level community composition. (**e**) Genus-level community composition. Asterisks indicate significant differences between treatments (*p* < 0.05, Duncan’s test). Values are means ± SE (*n* = 3). FB1: 27.0 g a.i. ha^−1^ florpyrauxifen-benzyl; FB2+S: 13.5 g a.i. ha^−1^ florpyrauxifen-benzyl + 0.09% (*v*/*v*) Sijiling.

Soil microbial diversity in the FB1 (florpyrauxifen-benzyl alone) and FB2+S (florpyrauxifen-benzyl + Sijiling adjuvant) treatments was analyzed 21 DAT to assess treatment impacts on soil microorganisms. Sequencing depth adequacy was confirmed by Shannon–Wiener and rank abundance curves, which stabilized after initial ascent (Supplementary Figure S5). Operational taxonomic unit (OTU) analysis revealed substantially higher microbial richness in FB2+S (7850.67 OTUs) compared to FB1 (3573.67 OTUs). Community richness, characterized by the Chao index, demonstrated significantly greater microbial abundance in FB2+S than FB1 (*p* < 0.05) (Figure 5a). Similarly, community diversity assessed via the ACE index showed significantly enhanced microbial richness in FB2+S relative to FB1 (*p* < 0.05) (Figure 5b).

Cluster analysis identified only four microbial groups playing significant roles in FB1, compared to sixteen functionally important groups in FB2+S (Figure 5c). Microbial composition analysis revealed similar dominant phyla (Proteobacteria, Bacteroidetes, Acidobacteria, Plantomycetes) in both treatments, but with distinct relative abundances. While Proteobacteria represented the most abundant phylum in both treatments, Bacteroidetes constituted the second most prevalent phylum in FB1, whereas Acidobacteria occupied this position in FB2+S (Figure 5d). Correspondingly, at the species level, *Luteitalea pratensis* (Acidobacteria) demonstrated greater prominence in FB2+S (Figure 5e).

### 2.5. Integrated Benefits Converge to Increase Rice Yield, with Soil Available Potassium and Organic Carbon as Key Drivers

**Figure 6:**
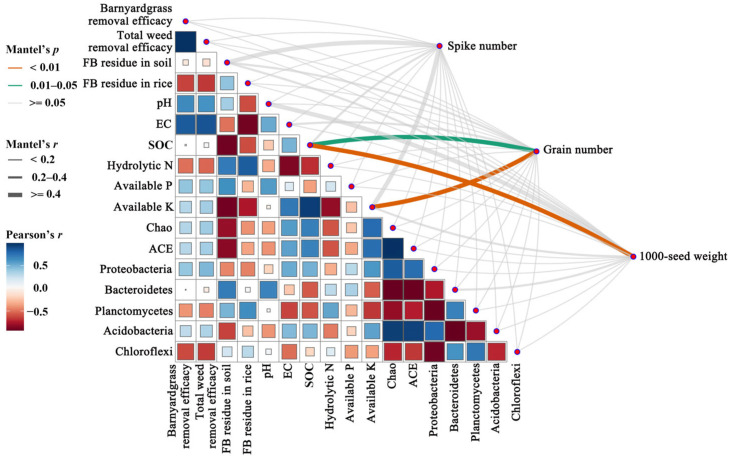
Mantel analysis of the correlation between rice yield differences and various factors. FB: florpyrauxifen-benzyl. In this figure, the line width connecting rice yield indicators (right) to environmental factors (left) is proportional to the Mantel statistic (r), with wider lines indicating stronger correlations. The line color represents statistical significance: green lines indicate significant correlations at *p* < 0.05, while orange lines indicate highly significant correlations at *p* < 0.01.

At harvest, FB2+S produced the highest yield components—71.67 productive panicles, 76.90 grains per panicle, and 28.34 g 1000-grain weight—significantly exceeding FB2 (*p* < 0.01) in all parameters and FB1 in grains per panicle (Figure 2e,f). Final yield reached approximately 0.62 kg/m^2^, significantly outperforming both FB1 and FB2. We synthesized various factors in this study to analyze why rice in the FB2+S treatment had a higher yield, especially in terms of the number of grains per panicle. We utilized the Mantel analysis method to integrate the 21-day measurement values of 17 factors, including barnyardgrass control efficacy, total weed control efficacy, residual amount in the soil, residual amount in rice, pH, EC, SOC, hydrolytic nitrogen, available phosphorus, available potassium, Chao index, ACE index, *Proteobacteria*, *Bacteroidetes*, *Planctomycetes*, *Acidobacteria*, and *Chloroflexi*, and analyzed the differential impact on rice yield indicators, including the number of productive panicles, number of grains per panicle, and 1000-grain weight (dependent variables) (Figure 6). FB2+S produced significantly more grains per panicle than FB1 and was strongly correlated with the higher available potassium content in the soil in FB2+S (*p* < 0.01) and the higher SOC content (*p* < 0.05).

## 3. Discussion

### 3.1. Adjuvant-Enabled Herbicide Dose Reduction Maintains Weed Control Efficacy

Our results demonstrate that adjuvant technology can effectively reduce florpyrauxifen-benzyl input by 50% without compromising barnyardgrass control. The excessive use of herbicides has consistently raised significant concerns due to its implications for increased agricultural production costs and environmental pollution [18,19]. A pervasive phenomenon in modern agriculture involves farmers applying high herbicide dosages in single applications to combat herbicide-resistant weeds, despite the availability of integrated weed management strategies. This practice persists even as researchers have developed various approaches to minimize herbicide application [20,21], with chemical control remaining the preferred method among farmers [22].

In Chinese rice production systems, barnyardgrass (*Echinochloa crus-galli*) represents the most detrimental weed species, traditionally controlled using herbicides such as propanil, penoxsulam, and quinclorac [7,23]. However, prolonged and excessive use has led to severe resistance development, particularly to quinclorac [1], rendering these herbicides increasingly ineffective. Florpyrauxifen-benzyl has emerged as a highly effective herbicide for barnyardgrass control, but its overuse raises concerns regarding rice safety, resistance evolution, and environmental impacts [24,25,26].

To address these challenges, this study investigated the potential of adjuvant technology to reduce florpyrauxifen-benzyl application rates, building upon the established success of adjuvants in enhancing herbicide performance [16,27,28]. Through comprehensive laboratory and field experiments, we demonstrated that the compound adjuvant Sijiling—composed of nonionic and anionic surfactants—exhibits remarkable synergistic effects with florpyrauxifen-benzyl, enabling a 50% reduction in herbicide dosage while maintaining equivalent efficacy against barnyardgrass. This reduction aligns with the goals of sustainable paddy rice production by lowering chemical input without compromising weed control. Importantly, this synergistic effect demonstrated specificity, as it was not universally applicable to other herbicide combinations (Supplementary Figure S4), highlighting the importance of tailored adjuvant–herbicide partnerships and suggesting that the observed benefits are not merely due to generic surfactant effects but involve specific physicochemical interactions.

### 3.2. Mechanisms of Synergy: Cuticular Wax Disruption and Hormonal Amplification

The present findings reveal that Sijiling enhances florpyrauxifen-benzyl efficacy through cuticular wax disruption and amplified phytohormone biosynthesis. The mechanism underlying compound adjuvant synergy primarily involves reducing the surface tension of spray droplets on plant leaves, thereby enhancing herbicide absorption [29,30]. The epicuticular wax layer serves as a crucial barrier against external stresses, including pesticide penetration [31,32,33,34]. Given that barnyardgrass has evolved sophisticated wax layer biosynthesis pathways as environmental adaptations [35], we investigated wax layer alterations following adjuvant application. Our SEM observations confirmed that Sijiling significantly enhanced florpyrauxifen-benzyl’s ability to disrupt the barnyardgrass wax layer (Figure 3), facilitating improved herbicide penetration through this primary defense barrier—a finding with implications for reducing the effective dose required for control.

As an auxin analog, florpyrauxifen-benzyl initiates complex signal transduction in sensitive plants [5], with ethylene and abscisic acid biosynthesis representing crucial downstream responses that constitute the direct cause of phytotoxicity [36,37,38]. The ethylene precursor ACC serves as an accurate indicator of ethylene synthesis intensity [1,39]. Our results show that barnyardgrass treated with florpyrauxifen-benzyl at 18 g a.i. ha^−1^ plus Sijiling accumulated significantly higher ACC and ABA concentrations than plants treated with florpyrauxifen-benzyl alone at either 18 or 36 g a.i. ha^−1^ (Table 2). This physiological potentiation confirms that Sijiling genuinely enhances herbicidal effectiveness at the biochemical level, enabling dose reduction while maintaining or even accelerating phytotoxic responses.

### 3.3. Reduced Herbicide Residues and Improved Soil Physicochemical Properties

Our study showed that adjuvant-assisted dose reduction significantly lowered florpyrauxifen-benzyl residues in both rice plants and soil, with faster degradation (Table 3). This finding is important because high herbicide residues can disrupt soil microbial functions and potentially affect plant growth [40]. This reduction in residue load likely alleviates long-term stress on soil biota and root health, contributing to the observed improvements in soil functioning and crop performance.

Soil physicochemical properties are fundamentally linked to soil health and crop productivity [12]. Our investigation revealed that the Sijiling and low-dose florpyrauxifen-benzyl combination (FB2+S) modified several soil parameters compared to high-dose herbicide treatment (FB1), including reduced hydrolytic nitrogen, increased soil organic carbon (SOC), and elevated available potassium (Figure 4). Hydrolytic nitrogen, essential for plant growth, helps maintain normal metabolic activity under adverse conditions [41,42,43]. A plausible explanation for the observed reduction in soil hydrolytic nitrogen is that enhanced herbicide efficacy induced stress responses, prompting accelerated nitrogen uptake by the plants. The dynamics of SOC and available potassium in relation to herbicide applications are complex [44], potentially involving direct herbicide effects or microbially mediated processes. Nevertheless, increases in these parameters benefit rice production, as SOC critically influences soil fertility and crop productivity [12,45], while potassium improves water use efficiency, photosynthesis, and yield [46,47,48].

### 3.4. Soil Microbial Community Shifts Under Reduced Herbicide Input

The reduced herbicide input achieved through adjuvant synergy enhanced soil microbial diversity and enriched beneficial taxa. Soil microorganisms drive nutrient cycling and serve as vital indicators of soil health, influencing root development, nutrient acquisition, and disease resistance [13]. The significantly higher Chao and ACE indices in FB2+S (*p* < 0.05) indicate that reducing herbicide input fosters a more diverse soil microbial community (Figure 5a,b). Such enhanced diversity is known to support more efficient nutrient cycling and energy flow, which can positively influence plant growth [49,50,51]. This is consistent with the observed improvements in soil organic carbon and available potassium (Figure 4c,f), suggesting a positive feedback loop between reduced chemical input, microbial recovery, and soil functioning.

LEfSe analysis identified more biomarker microorganisms in FB2+S (Figure 5c), indicating richer microbial community functionality [52,53]. Furthermore, FB2+S contained more Acidobacteria, which contribute to organic matter decomposition, nutrient cycling, and plant growth promotion [54]. Correspondingly, *Luteitalea pratensis* (Acidobacteria) was significantly more abundant in FB2+S (Figure 5e) [55]. The relatively higher Bacteroidetes proportion in FB1 (Figure 5d) may influence nitrogen cycling [56], potentially explaining the differential hydrolytic nitrogen patterns observed (Figure 4d). These findings indicate that reducing herbicide input through adjuvant synergy not only sustains paddy weed control but also fosters a more diverse and functionally rich soil microbial community. Given the well-established links between soil biodiversity and plant nutrient acquisition [13], such shifts may partly explain the increased rice yield observed in the FB2+S treatment.

### 3.5. Enhanced Rice Yield Is Linked to Improved Soil Available Potassium and Organic Carbon

The ultimate objective of adjuvant-assisted herbicide optimization is maintaining or improving crop yields while reducing production costs and environmental impacts. Yield improvements in the herbicide-treated plots relative to the non-treated controls primarily reflected effective weed competition elimination (Figure 2a,b). However, under comparable weed control efficacy, FB2+S produced significantly higher yields than FB1 (Figure 2f), with particularly notable improvements in grains per panicle.

The Mantel test analysis [57,58] provided mechanistic insight into this yield advantage: grains per panicle showed significant correlation with SOC (*p* < 0.05) and a strong correlation with available potassium (*p* < 0.01) (Figure 6). These correlations align with established knowledge—SOC is a key determinant of soil quality and crop productivity [59,60], while potassium is essential for stomatal regulation, enzyme activation, and assimilate transport in rice, directly influencing grain filling and yield [61,62,63]. The observed SOC–microbial structure interrelationship (Figure 5) further suggests that the enhanced microbial diversity in FB2+S may contribute to improved soil functioning, ultimately benefiting grain yield. As a high-potassium-demanding crop (150–300 kg ha^−1^ per season) with requirements comparable to nitrogen [1,64], the rice likely benefitted from the potassium-mediated stress tolerance enhancement in FB2+S [61,65].

### 3.6. Implications for Paddy Weed Management and Sustainable Rice Production

This study demonstrates that the compound adjuvant Sijiling acts as a potent synergist for florpyrauxifen-benzyl, enabling a 50% herbicide dose reduction while maintaining effective barnyardgrass control in paddy fields. The synergistic mechanism involves adjuvant-mediated disruption of the leaf cuticular wax barrier, enhancing herbicide penetration, and subsequent amplification of auxin-induced phytotoxic hormone biosynthesis. Importantly, this optimized chemical input not only reduces herbicide residues in rice and soil but also improves soil health indicators—including organic carbon, available potassium, and microbial diversity—ultimately contributing to increased grain yield. The strong links we observed between soil property improvements and yield gains highlight the importance of considering plant–soil feedbacks when designing reduced-input weed management strategies.

These findings provide a science-based strategy for herbicide dose optimization through adjuvant synergy, with direct implications for sustainable weed management in rice production systems. It is worth noting that herbicide dose reduction, if not properly implemented, carries the risk of sub-lethal dosing, which has been reported to promote recurrent selection and accelerate resistance evolution in *Echinochloa* species [66]. Moreover, the widespread occurrence of non-target-site resistance (NTSR) mechanisms, particularly enhanced metabolic detoxification in barnyardgrass populations [24], further underscores the importance of maintaining full herbicidal efficacy in any dose reduction strategy. The adjuvant-enabled approach demonstrated here mitigates these concerns by preserving effective weed control at the reduced dose. Future research should investigate the long-term effects of this reduced-input strategy on soil ecosystem services, explore its applicability to other herbicide–crop systems, and assess its economic viability at a farm scale. The integration of chemical, plant physiological, and soil biological perspectives demonstrated here offers a template for developing management practices that balance agricultural productivity with soil health protection.

## 4. Materials and Methods

### 4.1. Chemicals and Plant Materials

Florpyrauxifen-benzyl (3% EC, Rinskor^®^) was supplied by Corteva Agriscience (Indianapolis, IN, USA). The compound adjuvant Sijiling, provided by Sinon Chemical China Co., Ltd. (Shanghai, China), contains two active ingredients (Supplementary Figure S6): polyoxyethylene alkylaryl ether, a nonionic surfactant [67], and sodium dialkyl sulfosuccinate, an anionic surfactant [68]. Additional adjuvant specifications are in Supplementary Table S1.

Barnyardgrass seeds collected from paddy fields in Jinshan District, Shanghai (30°48′48.2″ N, 121°10′43.8″ E) in October 2020 and stored at −20 °C after drying were used for the herbicide/adjuvant efficacy evaluation. Seeds of the japonica rice cultivar ‘Nanjing 46’ served for the selectivity assessment.

### 4.2. Determination of the Optimal Sijiling Adjuvant Concentration

Twenty barnyardgrass seeds were sown in plastic pots (7 × 7 × 7 cm) filled with middle-loam soil collected from farmland in suburban Shanghai where no herbicides had been applied for over five years. The plants were grown in a controlled-environment chamber at 30/25 °C with a 14 h photoperiod (20,000 lx) and 10 h dark cycle, and watered every three days. After thinning to 10 uniform plants per pot, barnyardgrass was cultivated to the 3.0–4.0 leaf stage for herbicide application. The experimental design included one control group (CK, untreated) and seven treatment groups (T1–T7). All the treatments received florpyrauxifen-benzyl at 3.6 g a.i. ha^−1^, while the adjuvant Sijiling was applied at 0, 0.015, 0.03, 0.045, 0.06, 0.075, 0.09, and 0.105% (*v*/*v*) of the spray solution. Applications were made using an HCL2000 walking-type sprayer equipped with a fan-shaped nozzle, delivering 50 mL per treatment (450 L ha^−1^ water volume) at 35 cm height and 240 mm/s travel speed. After the spray droplets dried, the plants were maintained in a greenhouse for 21 days before harvesting for fresh weight measurement. The inhibition rate of aboveground fresh weight was calculated relative to the control. The experiment employed four biological replicates and was conducted twice. The same methodology was applied to evaluate the potential synergistic effects of the selected adjuvant with other herbicides.

### 4.3. Dose–Response Bioassay for Florpyrauxifen-Benzyl with and Without Sijiling

An additional whole-plant bioassay was conducted to determine the optimal florpyrauxifen-benzyl dosage and its residual effect when combined with the optimal Sijiling adjuvant concentration (0.09% *v*/*v*). Plant cultivation and spraying followed the methods described above. Two treatment series were established: FB (florpyrauxifen-benzyl alone at 0, 0.5625, 1.125, 2.25, 4.5, 9.0, 18.0, and 36.0 g a.i. ha^−1^) and FB+S (identical florpyrauxifen-benzyl doses with Sijiling). After 21 days, aboveground fresh weight inhibition rates were calculated. The experiment included four biological replicates and was repeated twice. GR_50_ values for plant height inhibition were determined using a four-parameter logistic model in R 4.1.3 with the “drc” package (version 3.0-1) [69,70].

Y=c+{(d−c)/(1+exp (b(log x−log e)))}

Parameter e is also denoted as GR_50_ and represents the dose producing a response halfway between the upper limit, d, and the lower limit, c. Parameter b denotes the relative slope around e.

### 4.4. Field Validation of Herbicide Reduction Efficacy

A paddy field experiment was conducted in Fengxian District, Shanghai (30°53′39.9″ N, 121°23′30.3″ E), China, in a field naturally infested with *Echinochloa* spp., *Ammannia* spp., and *Cyperus difformis*. Four treatments were established: FB1 (27.0 g a.i. ha^−1^), FB2 (florpyrauxifen-benzyl at 13.5 g a.i. ha^−1^), FB2+S (13.5 g a.i. ha^−1^ + 0.09% *v*/*v* Sijiling adjuvant), and an untreated control (CK). The experiment employed a completely randomized design with twelve 20 m^2^ plots (5 m × 4 m) and three replicates per treatment. Herbicides were applied at the rice 4.0–5.0 leaf stage (28 days after sowing) using an HD 900 electric sprayer (Agrolex, Singapore) equipped with a fan-shaped nozzle. Each plot received 900 mL of spray solution at 230.0 kPa pressure. Applications were made under cloudy conditions in 2021 (27.1 °C, 59.5% humidity, 1.1 m/s wind speed) and 2022 (29.1 °C, 59.6% humidity, 1.2 m/s wind speed).

At 14 days after treatment (DAT), ten rice plants per plot were sampled to measure tiller number, plant height, and fresh weight. Weed control efficacy was assessed at 21 DAT by counting the remaining barnyardgrass and other weeds within four randomly placed 0.25 m^2^ quadrats per plot. At harvest (October), yield components including effective panicles, grains per panicle, and 1000-grain weight were determined from four 0.25 m^2^ quadrats per plot. The panicles were dried to constant weight, and 1000-grain weight was measured using a precision balance (ME2002E, Mettler-Toledo, Greifensee, Switzerland). All the data were analyzed using SPSS Statistics 20.0 with Duncan’s multiple range test (*p* < 0.05).

### 4.5. Scanning Electron Microscopy (SEM) of Leaf Cuticular Wax

Barnyardgrass plants at the 2.5–3.5 leaf stage were treated with: water (control), florpyrauxifen-benzyl (13.5 g a.i. ha^−1^), florpyrauxifen-benzyl (27.0 g a.i. ha^−1^), or florpyrauxifen-benzyl (13.5 g a.i. ha^−1^) plus Sijiling adjuvant (0.09% *v*/*v*). Leaf samples (1–2 mm^3^) were collected 12 h post-treatment and immediately fixed in 2.5% glutaraldehyde at 4 °C overnight. Following primary fixation, the samples underwent rinsing with 0.1 M phosphate buffer (pH 7.0), secondary fixation with 1% osmium tetroxide, and progressive dehydration through an ethanol series (30–100%). Subsequent processing included treatment with isoamyl acetate, critical point drying, and sputter-coating before examination using scanning electron microscopy (SEM).

### 4.6. Quantification of Abscisic Acid (ABA) and 1-Aminocyclopropane-1-carboxylic Acid (ACC)

Whole barnyardgrass plants at the 2.5–3.5 leaf stages were sprayed with 13.5 g a.i. ha^−1^ of florpyrauxifen-benzyl, 27.0 g a.i. ha^−1^ of florpyrauxifen-benzyl, or 13.5 g a.i. ha^−1^ of florpyrauxifen-benzyl + Sijiling (0.09%, *v*/*v*). After 0 and 12 h, shoots were collected and immediately frozen in liquid nitrogen. The sample processing methods and test protocol are described in the Supplementary Materials. All ACC or ABA content data for the two treatments were subjected to significance analysis using SPSS Statistics (for Windows, Version 20.0; IBM, Armonk, NY, USA) using Duncan’s multiple range test (*p* < 0.05).

### 4.7. Analysis of Florpyrauxifen-Benzyl Residues in Rice and Soil

On days 0 (the day after herbicide treatment), 3, 7, 14, and 21 after herbicide treatment, rice and soil samples from the FB1 and FB2+S experimental plots (described in Section 4.4) were collected and quickly placed in sampling boxes containing ice packs and brought back to the laboratory for herbicide residue testing. Three replicates were performed for each treatment group. The details of the detection are provided in the Supplementary Materials. The three detected duplicate data for each treatment were converted into a percentage relative to the data on day 0, and then the time for decomposition of 50% florpyrauxifen-benzyl was calculated using the R software.

### 4.8. Assessment of Soil Physicochemical Properties

Soil samples were collected from FB1 (27.0 g a.i. ha^−1^ florpyrauxifen-benzyl) and FB2+S (13.5 g a.i. ha^−1^ + 0.09% Sijiling) plots at 0, 3, 7, 14, and 21 days after treatment. Samples were immediately transported on ice for analysis of pH (electrometric method, NY/T 1121.2-2006) [71], electrical conductivity (HJ 802-2016) [72], soil organic carbon (potassium dichromate oxidation, NY/T 1121.6-2006) [73], hydrolytic nitrogen (alkali extraction volumetric method, LY/T 1228-2015) [74], available phosphorus (sodium bicarbonate extraction with molybdenum antimony anti-colorimetry, NY/T 1121.7-2014) [75], and available potassium (ammonium acetate extraction with flame photometry, LY/T 1234-2015) [76]. All treatments were replicated three times in two independent experiments. Data were analyzed by ANOVA and Duncan’s test (*p* < 0.05) using SPSS Statistics 20.0.

### 4.9. Soil Microbial Community Analysis by High-Throughput Sequencing

On day 21 after herbicide treatment, soil samples were collected from the FB1 and FB2+S experimental plots (described in Section 4.4). Samples were quickly stored in liquid nitrogen and immediately subjected to soil microbial library construction and sequencing. The specific methods for sequencing, as well as the analysis methods for microbial abundance, richness, and structural differences, are shown in Supplementary Material.

### 4.10. Statistical Analysis of Yield Determinants (Mantel Test)

The Mantel test was used to evaluate the relationship between rice yield indicators and soil environmental factors. This nonparametric method is well suited for comparing two distance matrices and was therefore selected to assess the correlations between the yield-related matrix (productive panicles, grains per panicle, and 1000-grain weight) and the matrix of soil physicochemical properties, microbial diversity indices, and weed control efficacy. The analysis was performed using the “vegan” package (version 2.6-4) in R software (version 4.1.3) [70]. Two levels of correlation were set, with *p* < 0.05 indicating a significant correlation and *p* < 0.01 indicating a highly significant correlation.

### 4.11. Ethical Approval and GenAI Use Statement

This study did not involve animals or human subjects, and therefore no ethical approval was required. No generative artificial intelligence (GenAI) tools were used for data generation, analysis, or manuscript writing in this study.

## 5. Conclusions

This study demonstrates that the compound adjuvant Sijiling enables a 50% reduction in florpyrauxifen-benzyl application while maintaining effective control of the dominant paddy weed barnyardgrass (*Echinochloa crus-galli*). The synergistic mechanism is driven by the adjuvant-mediated disruption of the leaf cuticular wax barrier, which amplifies the biosynthesis of the phytotoxic hormones ethylene and ABA. Beyond sustaining paddy weed control, this reduced-rate strategy delivers tangible co-benefits for the plant–soil system: it lowers herbicide residues in rice and soil, increases soil organic carbon and available potassium, and fosters a more diverse and functionally rich microbial community, notably enriching beneficial Acidobacteria. These improvements in soil health are directly linked to significant increases in grain yield, particularly grains per panicle, as confirmed by Mantel analysis. Our findings provide a science-based framework for optimizing herbicide use in paddy rice systems through targeted adjuvant synergy, demonstrating that reductions in chemical inputs can be reconciled with enhanced soil ecosystem function and crop productivity. This strategy offers a practical and sustainable approach to integrated weed management in paddy fields, contributing to the long-term sustainability of rice production.

## 6. Patents

A patent directly resulting from the work reported in this manuscript has been granted: Gao, Y.; Shen, G.; Tian, Z.; Yuan, G.; Fang, J. Herbicidal composition for controlling resistant weeds and application thereof. Chinese Patent ZL 2023 1 0221884.2, 22 April 2025.

## Figures and Tables

**Table 1:** The impact of candidate adjuvants on the control efficacy of florpyrauxifen-benzyl on barnyardgrass.

Adjuvants	FB-High	FB2-Low	FB-Low+V1	FB-Low+V2	FB-Low+V3
Huoniu	80.26 ^abcde^	68.52 ^gh^	56.59 ^i^	56.79 ^i^	45.24 ^k^
Maisi			64.82 ^h^	65.62 ^h^	72.72 ^efgh^
Hasuteng			47.28 ^jk^	54.10 ^ij^	65.35 ^h^
Jiadeli			75.41 ^cdefg^	71.40 ^fgh^	71.96 ^fgh^
Meidun			74.67 ^cdefg^	76.29 ^bcdefg^	83.79 ^ab^
Sijiling			73.74 ^defg^	82.15 ^abc^	84.51 ^a^
Jijian			81.26 ^abcd^	77.27 ^abcdef^	55.95 ^i^

FB-high and -low: 18.0 and 9.0 g a.i. ha^−1^ of florpyrauxifen-benzyl; V1–V3: minimum, median, and maximum recommended adjuvant addition ratios. Different letters indicate significant differences (Duncan’s test, *p* < 0.05). Specific information on adjuvants can be found in Supplementary Table S1.

**Table 2:** ACC and ABA contents at 12 h after different treatments.

Indicator	Control	FB1	FB2	FB2+S
ACC (ng g^−1^)	25.97 ± 0.69 d	1606.77 ± 66.46 b	534.39 ± 10.97 c	2404.17 ± 43.03 a
ABA (ng g^−1^)	11.47 ± 0.28 c	51.99 ± 1.52 b	49.44 ± 1.74 b	76.93 ± 1.63 a

FB1 and FB2: 27.0 and 13.5 g a.i. ha^−1^ florpyrauxifen-benzyl; FB2+S: 13.5 g a.i. ha^−1^ florpyrauxifen-benzyl + 0.09% (*v*/*v*) Sijiling. Different letters within a row indicate significant differences (Duncan’s test, *p* < 0.01).

**Table 3:** Residual amounts of florpyrauxifen-benzyl in rice and soil.

Treatment	Residual Amounts (ppb) at Different Times					Half-Life (DT50, Days)
	0 Days	3 Days	7 Days	14 Days	21 Days	
FB1 in rice	697.49 ± 11.00 A	596.81 ± 7.32 B	267.08 ± 6.23 E	243.17 ± 4.46 EF	11.39 ± 1.81 H	6.45
FB2+S in rice	484.12 ± 12.38 C	359.94 ± 4.88 D	216.95 ± 6.18 FG	189.41 ± 2.19 G	5.63 ± 0.15 H	6.59
FB1 in soil	25.34 ± 0.37 a	16.53 ± 0.58 b	10.79 ± 0.26 c	6.45 ± 0.24 d	3.46 ± 0.10 e	5.29
FB2+S in soil	15.12 ± 0.43 b	7.11 ± 0.27 d	3.04 ± 0.05 e	2.52 ± 0.11 e	2.32 ± 0.07 e	2.34

Values are means ± SE (*n* = 3). Uppercase letters (A–H) compare temporal differences between treatments within rice samples; lowercase letters (a–e) compare temporal differences within soil samples. Different letters within the same case group indicate significant differences (Duncan’s test, *p* < 0.05).

## Data Availability

The microbial diversity sequencing data generated in this study have been deposited in the NCBI database under accession number PRJNA1100743. All other data supporting the reported results are available from the corresponding author upon reasonable request.

## Supplementary Materials

The following supporting information can be downloaded at: https://www.mdpi.com/article/10.3390/plants15111688/s1, Figure S1: Deformity of rice stem caused by excessive use of florpyrauxifen-benzyl; Figure S2: Soybean planted adjacent to rice field was damaged by florpyrauxifen-benzyl; Figure S3: Effects of candidate adjuvants on the control of barnyard grass by florpyrauxifen-benzyl; Figure S4: The impact of the adjuvant, Sijiling on weed control of other commonly used herbicides in rice field; Figure S5: Microbial sequencing depth of all samples; Figure S6: The product label of the adjuvant “Sijiling”; Table S1: Category and source information of all candidate adjuvants.
