# Supplementary material for: Adjuvant-Enabled Halving of Florpyrauxifen-Benzyl Dose Maintains Paddy Weed Control and Enhances Soil Health and Rice Yield
Source: Plants (Basel). 2026 May 29;15(11):1688. doi: 10.3390/plants15111688 (PMC13259329; doi:10.3390/plants15111688)
Supplement: Supplementary file 1 [file plants-15-01688-s001.zip › plants-4299471-supplementary.pdf]

Adjuvant-Enabled Halving of Florpyrauxifen-Benzyl Dose Maintains Paddy Weed Control and Enhances Soil Health and Rice Yield

Yuan Gao [1,*], Huifeng Wang [2], Jiapeng Fang [1], Guohui Yuan [1], Zhihui Tian [1], Lirong Wang [2]

[1] Eco-Environmental Protection Research Institute, Shanghai Academy of Agricultural Sciences, Shanghai 201403, PR China; gaoyuan@saas.sh.cn, 20220701@saas.sh.cn, yuanguohui@saas.sh.cn, tianzhihui@saas.sh.cn

[2] Faculty of Chemical Engineering and Energy Technology, Shanghai Institute of Technology, Shanghai 201418, PR China; hfwang@sit.edu.cn, 256061130@sit.edufp.cn

* Correspondence: gaoyuan@saas.sh.cn

[*] Corresponding author.

E-*mail address*: hfwang@sit.edu.cn (H. Wang).

tianzhihui@saas.sh.cn (Z. Tian)

ACC content determination method

A sample of each plant was ground into powder in liquid nitrogen, accurately weighed (0.2 g), and transferred to a test tube. ddH_2O, which was precooled to 4 °C, was then added to the test tube and allowed to stand for 2 h for extraction. The supernatant was retained after centrifugation twice at 10,000 ×*g* for 5 min at 4 °C, after which, the two extracted supernatants were combined, passed through an McX column, and washed with 5 mL of water. The eluent was analyzed using high-performance liquid chromatography–tandem mass spectrometry (HPLC–MS/MS) after passing through a 0.22-μM filter membrane.

For the HPLC analysis using a reversed-phase chromatography column (Poroshell 120 SB-C18, 2.1 × 150, 2.7 μm), the injection volume was 2 μL, and the column temperature was set to 35 °C. The mobile phase used was A: B = acetonitrile: (water/0.1% formic acid) = 3:7. The elution gradients were equal. The electrospray ionization (ESI) source and positive ion modes (+4500 V) were used for the spectrometry analysis. The temperature was set to 350 °C, the curtain air was set to 15 PSI, the atomization air pressure was set to 65 PSI, and the auxiliary air pressure was set to 70 PSI. The other testing conditions are listed in the following table.

**Substance**	**Polarity**	**Parent Ion (m/z)**	**Daughter Ion (m/z)**	**De Clustering Voltage (V)**	**Collision Energy (V)**
ACC	+	102.0	55.9 */84.0	40	19/42

* Quantitative ion.

ABA content determination method

A single sample was ground into a powder in liquid nitrogen, accurately weighed (0.5 g), and transferred to a test tube. Ten times the volume of acetonitrile solution

and 4 μL of the internal standard mother liquor were added. The solution was extracted at 4 °C overnight and centrifuged at 12000 ×*g* for 5 min, and the supernatant was collected. Five times the volume of the acetonitrile solution was added to the precipitate for extraction, and the supernatants obtained from the two extractions were combined. Subsequently, 15–40 mg of C18 filler was added to the supernatant. After violent shaking for 30 s, the mixture was centrifuged at 10000 ×*g* for 5 min, and the supernatant was collected. The supernatant was dried under nitrogen and added to 400 μL of methanol. The solution was then passed through a 0.22-μM organic phase filter membrane and stored at - 20 °C for detection.

For the HPLC analysis using a reversed-phase chromatographic column (Poroshell 120 SB-C18, 2.1 × 150, 2.7 μm), the injection volume was 2 μL, and the column temperature was set to 30 °C. The mobile phase was A: B = (methanol / 0.1% formic acid): (water / 0.1% formic acid). The elution gradients are listed in the following table.

Time (min)	Flow velocity (mL/min)	A%
0-1	0.3	20
1-3	0.3	Increase from 20 to 50
3-9	0.3	Increase from 50 to 80
9-10.5	0.3	80
10.5-10.6	0.3	Decrease from 80 to 20
10.6-13.5	0.3	20

For the spectrometry analysis, ESI and positive and negative ion modes (+4500 V, -4000 V) were used. The scan type was MRM. The temperature was set to 400 °C, the curtain air was set to 15 PSI, the atomization air pressure was set to 65 PSI, and the auxiliary air pressure was set to 70 PSI. The other testing conditions are listed in the following table.

Substance	Polarity	Parent ion (m/z)	Daughter ion (m/z)	De clustering voltage (V)	Collision energy (V)
ABA	-	263.1	153.1*/204.2	-60	-14/-27

*Quantitative ion

Residual amounts of florpyrauxifen-benzyl Detection method

1) Extraction of herbicide residues from rice plant

A total of 5 g of milled rice plants were accurately weighed and placed in a 250 ml Erlenmeyer flask, and then 10 ml of 0.1% formic acid-water aqueous solution was added and soaked for 30 min. Then, 20 ml of acetonitrile solution containing 0.1% formic acid by volume fraction was added, and the sealed flask was placed in a shaking box for 250 revolutions to extract for 1 hour. The extract was pumped and filtered into a stoppered measuring cylinder containing 2 g of sodium chloride, and fully shaken. After standing for layering, the supernatant was taken for purification.

2) Extraction of herbicide residues from soil

A total of 5 g of ground soil samples were accurately weighed and placed in a 100 ml centrifuge tube, followed by 20 ml of acetonitrile solution containing 0.1% formic acid by volume and 5 ml of water. Vortex mixer was used to vortex the mixture

for 5 min, and then it was vibrated by ultrasound for 10 min. After 2 g of sodium chloride was added, it was vortex vibrated for 3 min. The sample was centrifuged at 4000 rpm/min for 5 min, and then the supernatant was taken for purification.

3) Purification of supernatant

A total of 2 ml of plant or soil supernatant was transferred to a 4 ml centrifuge tube containing 50 mg Florisil soil purifier. The mixed liquid was swirled for 2 min and centrifuged at 4000 rpm/min for 2 min. then 1 ml of liquid was removed and blown to nearly dry with nitrogen. Chromatographic acetonitrile (1 ml) was used to determine the volume and filtered through a 0.22 μ mol L^{-1} membrane.

4) Determination of residual amount

Testing conditions

Ultra performance liquid chromatography/mass spectrometry: Waters ACQUITY H-Class PLUS；

Chromatographic column: Waters T3 analytical column, 2.1 mm×100 mm×1.8 μm;

Velocity of flow: 0.25 mL/min；

Column temperature: 30℃；

Sample size: 2.0 μL；

Retention Time: 2.90 min;

Mobile phase: acetonitrile + 0.1% formic acid-water (60:40, v/v).

Ionization mode	ESI+

Capillary voltage	3.35 KV
Source temperature	150 ℃
Desolvent temperature	500 ℃
Solvent removal airflow	Nitrogen, 600L/h
Collision Gas	Argon
Monitoring mode	Multiple reaction ion monitoring

Multiple reaction detection conditions

Residue	Ionization method ESI	Parent Ion m/z	Daughter Ion m/z	residence time /s	Cone voltage /V	Collision /eV	Retention /min
florpyrauxifen-benzyl	+	439.1	91.1* / 65.3	0.025	46	47 / 88	2.90

"*" mean quantitative ion

5) Calculation of residual amount

The following formula was used to calculate the residual amount of florpyrauxifen-benzyl. The calculation results deducted the values of the control check, and the measured results were represented by the arithmetic mean of parallel measurements, with two significant figures retained.

$$Xi = \frac{A \times Cs \times Vx \times Vex}{As \times Vri \times m}$$

The meaning of each letter in the formula is as follows. X*i*: Residue of analyte in sample, mg/kg; A: Peak area of analyte in sample; C*s*: Concentration of analyte in standard solution, mg/L; m: Quality of the test sample, g; V*ri*: Volume of separated liquid, mL; A*s*: Peak area of analyte in standard solution; V*ex*: Total volume of extract, mL; V*x*: Constant volume of sample, mL.

Soil microbial diversity detection and analysis methods

1) Library construction

The total DNA of the soil sample was extracted using EZ.N.A. ® Soil DNA Kit (X) Extract according to the instructions. The amplicons of the target bacterial population are prepared based on DNA obtained from tuple samples; the study on bacterial community structure was conducted by PCR amplification using 16s rRNA V1-V9 region primers 27F (upstream primer) 5 '- arcode- AGAGTTTGATCMTGGCTCAG) -3' and 1492R (downstream primer) 5 '- CRGYTACCTTGTTACGACTT-3'. The amplified product was purified by 2% agarose gel electrophoresis. The purified PCR product is accurately quantified using Qubit ® 3.0 (Life Technologies, Carlsbad, America), and then mixed in corresponding proportions according to the sequencing requirements of each sample. The mixed DNA products were used to construct PacBio sequencing library. The SMRTbell template was purified by magnetic beads after repairing and flattening the end reaction, connecting the connector to form a

dumbbell like structure, and removing the fragments of the unconnected connector by exonuclease. Finally, the purified product was passed through 1.2% agarose gel to obtain a 16S SMRTbell library with the expected size (about 1.5 kb) for sequencing on PacBio sequel IIe platform.

2). Optimization of the original sequence

The original sequence obtained by sequencing was subjected to quality control using SMRTLINK (V9). The restrictions are as follows: 1) the minimum number of sequencing cycles is 5; 2) the minimum prediction accuracy is 90; 3) the minimum insertion sequence length was 1400bp; 4) the maximum sequence length was 1800bp. In order to obtain high-quality subreads, the sequences are further filtered for quality using the following method: 1) polymerase reads shorter than 1000bp were removed; 2) reads with low quality values (Score value less than 0.8) are removed; 3) the linker sequence contained in polymerase reads has been removed.

3) Cluster processing of sequences

The OTU clustering method based on 98.65% similarity was used for amplicon analysis, which met the international default standard of full-length Strain level. Non repetitive sequences are extracted from optimized sequences in order to reduce redundant computation during the analysis process (http://drive5.com/usearch/manual/dereplication.html); single sequences without duplicates are also removed (http://drive5.com/usearch/manual/singletons.html). Next, OTU clustering was performed on non-repetitive sequences (excluding single sequences) based on 98.65% similarity, and chimeras were removed. Finally, all representative sequences of OTUs were obtained. All optimized sequences are mapped to OTU representative sequences, and the OTU table is generated after the sequences with a similarity of more than 98.65% with OTU representative sequences

are selected.

4) Taxonomic analysis

RDP classifier (http://rdp.cme.msu.edu/, version 2.2) is used to annotate the species classification of each sequence, according to the Silva 16S rRNA database (v138), with a threshold of 80%. The uclust algorithm is used for taxonomic analysis of OTU representative sequences, and the community composition of each sample is counted at the phylum level to obtain the species classification information corresponding to each OTU.

5) Diversity analysis

The Schao1 index calculation formula used for alpha analysis is as follows:

$$Schao1 = Sobs + n_1(n_1 - 1)/2(n_2 + 1)$$

Schao1, estimated number of OUT; Sobs, measured actual number of OUT; n1, the number of OTUs containing only one sequence (e.g. "singletons"); n2, the number of OTUs containing two sequences (e.g. "doubletons ").

The ACE index calculation formula used for alpha analysis is as follows:

$$ACE = S_{abund} + S_{rare}/C_{ace} + F1/C_{are}*\gamma^2_{ace}$$

S_{abund}, number of abundant (abundance threshold > n) species; S_{rare}, number of rare (abundance threshold < n) species; F_1, Number of species containing only one individual； γ^2_{ace}, Estimation of coefficient of variation for rare species.

Prior to alpha diversity calculation, the OTU table was rarefied to an even sequencing depth of 12,800 reads per sample to normalize for differences in sequencing depth across treatments.

6) Linear Discriminant Analysis Effect Sizes (LEfSe) analysis

First, the features with significant abundance differences in each treatment were detected using the non-parametric factorial Kruskal-Wallis（KW）sum-rank test. Then, groups with significant differences in abundance were identified and labeled. Linear discriminant analysis (LDA) was used to estimate the impact of each component (species) abundance on the difference effect. The linear discriminant analysis (LDA) threshold was set to 2.0 to identify significantly enriched microbial taxa between treatments.

Supplementary material Captions

Figures:

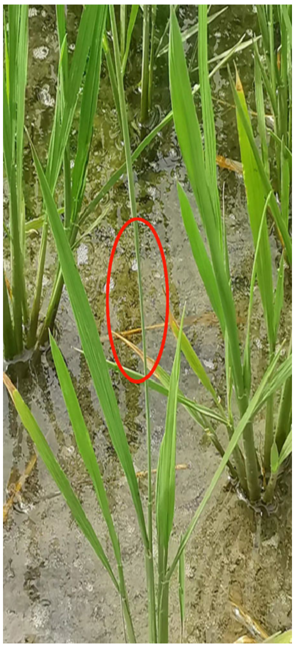

Figure S1. Deformity of rice stem caused by excessive use of florpyrauxifen-benzyl.

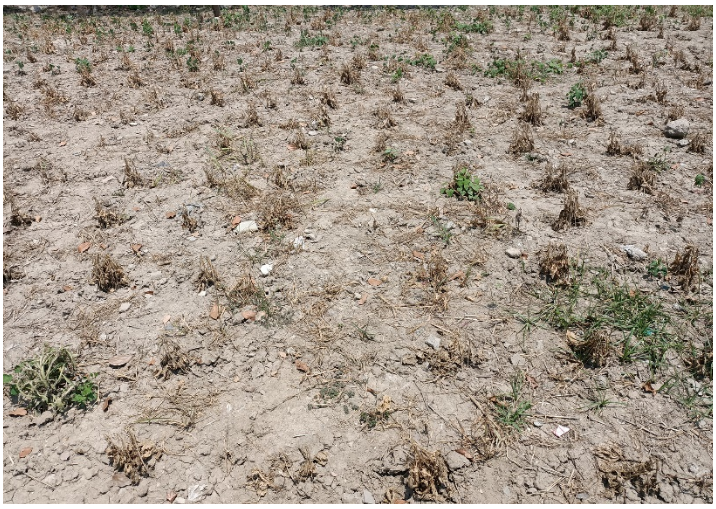

Figure S2. Soybean planted adjacent to rice field was damaged by florpyrauxifen-benzyl.

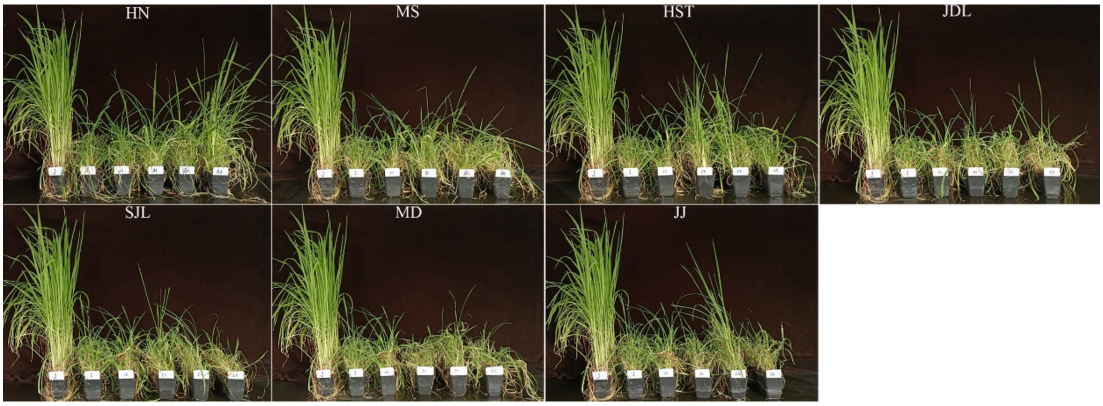

Figure S3. Effects of candidate adjuvants on the control of barnyard grass by florpyrauxifen-benzyl. HN: Huoniu, MS: Maisi, HST: Hasuteng, JDL: Jiadeli, SJL: Sijiling, MD: Meidun, JJ: Jijian. Details of these adjuvants are shown in supplementary table S1. From left to right in each figure are: control check, florpyrauxifen-benzyl dosage 1, florpyrauxifen-benzyl dosage 2, florpyrauxifen-benzyl dosage 2 + adjuvant v1, florpyrauxifen-benzyl dosage 2+adjuvant v2, florpyrauxifen-benzyl dosage 2 + adjuvant v3.

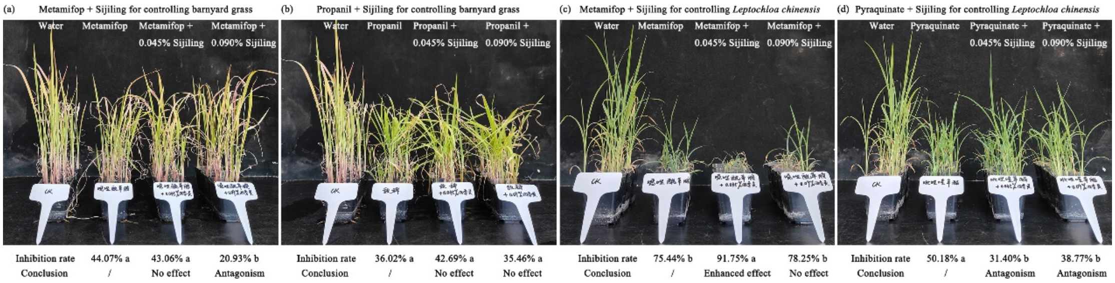

Figure S4. The impact of the adjuvant, Sijiling on weed control of other commonly used herbicides in rice field.

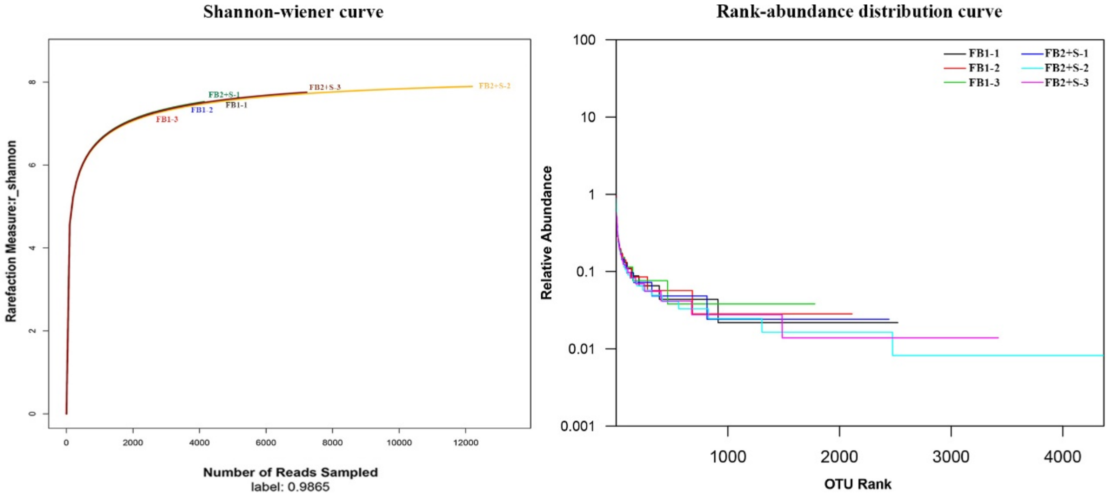

Figure S5. Microbial sequencing depth of all samples.

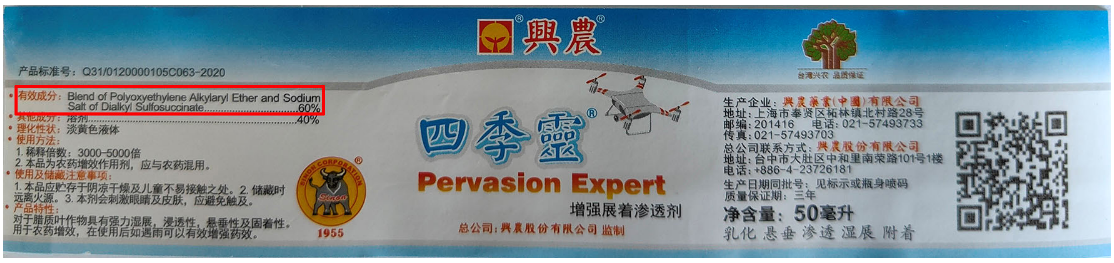

Figure S6. The product label of the adjuvant "Sijiling". The effective ingredients of this additive are circled in red.

Tables:

Table S1. Category and source information of all candidate adjuvants

Adjuvant	Category	Addition amount (V1, V2, V3 (ml ha^{-1}))	Manufacturers
Huoniu	Lecithin	750, 1125, 1500	Anhui Nongzhiyou Agricultural Materials Co., Ltd, Suzhou, China
Jiadeli		1500, 2250, 3000	AXEB BIOTECH S.L, Shanghai, China
Maisi	Plant oil	675, 900, 1125	Beijing Guangyuan Yinong Chemical Co., Ltd, Beijing, China
Hasuteng		450, 675, 900	Bayer CropScience China Co., Ltd, Hangzhou, China
Meidun		225, 300, 450	Sinon Chemical (China) Co., Ltd., Shanghai, China
Sijiling	Silicone	90, 112.5, 150	Sinon Chemical (China) Co., Ltd., Shanghai, China
Jijian	Nonionic surfactant+ Plant oil	180, 225, 270	Chengdu Jijian Biotechnology Co., Ltd., Chengdu, China
